# Thrombotic microangiopathy following chimeric antigen receptor T-cell therapy 

**DOI:** 10.5414/CNCS111045

**Published:** 2023-02-16

**Authors:** Matthew S. Wu, Abbal Koirala

**Affiliations:** 1 Department of Medicine, and; 2 Section of Nephrology, Department of Medicine, University of Washington Medical Center, Seattle, WA, USA

**Keywords:** thrombotic microangiopathy, CAR-T, transplant, endothelium

## Abstract

Introduction: Thrombotic microangiopathy (TMA) is characterized by microangiopathic hemolytic anemia and is associated with a variety of conditions and following hematopoietic stem cell transplantation. Chimeric antigen receptor T-cell (CAR-T) therapy is a novel immunotherapeutic approach using genetically modified autologous T cells. CAR-T therapy has been linked with injuries to vascular endothelium, but a direct association between CAR-T and TMA has not been reported. Case reports: Two cases of TMAs following CAR-T treatment are reported here. In each case, clinical evidence of kidney injury, thrombocytopenia, and hemolytic anemia became apparent 2 – 3 months following CAR-T infusion. We describe the clinical course, management, and outcome of these experiences. Discussion/Conclusion: CAR-T cell therapy-associated TMA (CAR-T TMA) appear to be an entity that shares overlapping clinical features with transplant-associated TMA (TA-TMA). Based on our preliminary clinical observations, we discuss the best clinical diagnosis/classification criteria, underlying pathophysiology, and the implication of the apparently self-limiting course. With increasing use of CAR-T cell treatment in hematologic malignancies, systematic studies will be necessary to improve management of CAR-T TMA.

## Introduction 

Thrombotic microangiopathy (TMA) is characterized by microthrombi formation resulting in microangiopathic hemolytic anemia (MAHA) and thrombocytopenia leading to tissue damage. The pathophysiology of TMA involves underlying endothelial injury and complement activation affecting the microvasculature. TMA is the dominant phenotype of thrombotic thrombocytopenic purpura (TTP) and hemolytic uremic syndrome (HUS), and can be elicited by pregnancy, malignancies, and autoimmune states [1]. In hematopoietic stem-cell transplant (HSCT), TMA has been primarily described in allogeneic stem cell transplantation, where it is increasingly associated with high-dose chemotherapy, total body irradiation, HLA mismatch, unrelated donor, graft-versus-host disease, and exposure to calcineurin inhibitors [2]. Transplant-associated TMA (TA-TMA) has also been reported in autologous transplant [3]. 

Chimeric antigen receptor (CAR)-T cell therapy is a rapidly evolving immunotherapeutic approach involving the use of ex-vivo expanded chimeric antigen receptor modified autologous T cells. Commonly observed toxicity uniquely associated with CAR-T therapy include the cytokine release syndrome (CRS) and immune effector cell-associated neurotoxicity syndrome (ICANS). These serious, and at times life-threatening, post-treatment conditions result from a milieu of inflammatory mediators, endothelial injuries, and complement activations, which overlaps with underlying pathophysiology of TMA. Yet, TMA has not been well described in CAR-T therapy. 

The present case study describes two patients treated with CAR-T cell therapy, subsequently presenting with kidney injury, thrombocytopenia, and MAHA consistent with TMA. 

## Case 1 

A 64-year-old male with relapsed diffuse large B-cell lymphoma received anti-CD19 CAR-T cell therapy in 12/2020. His initial treatment course was complicated by severe CRS resulting in spinal cord edema and chronic urinary retention. After 3 months of physical recovery, the patient was found to have rising serum creatinine peaking to 1.4 mg/dL in 3/2022, thrombocytopenia to a nadir of 10,000/μL, and progressive anemia requiring weekly transfusion. A bone marrow biopsy showed a consumptive process without morphologic dysplasia. Treatment of immune thrombocytopenic purpura with intravenous immune globulin (IVIG), steroid, and thrombopoietin receptor agonist romiplostim did not resolve his thrombocytopenia. Serum lactate dehydrogenase (LDH)was found to be > 400 units/L, haptoglobin < 30 mg/dL, and schistocytes demonstrated on peripheral blood smear (> 4/HPF). The nephrology service was consulted for MAHA and kidney injury. 

Urinalysis demonstrated 1+ protein, with no bacteria or casts present. An initial workup for immune-mediated TTP consisted of a negative ADAMST13 antibody screen. Direct antiglobulin (Coombs) test was negative thereby excluding autoimmune hemolytic anemia. Coagulation assays were found to be normal. Folate, B12, and methylmalonic acid levels were all within normal ranges. Autoimmune testing panels (ANA, ANCA) were unremarkable. Complement C3/C4/CH50 were similarly within normal limits. A send out Genetic and Complement Renal Panel to the University of Iowa, which tests for 13 genes implicated in hereditary disorders associated with atypical HUS and TMA, returned negative. All viral studies were negative. Patient remained normotensive. 

A diagnostic kidney biopsy was deferred given the patient’s marked thrombocytopenia and risk for bleeding. Fortunately, the patient improved over the next year with eventual recovery to baseline creatinine levels and transfusion independent platelet counts by 11/2021. 

## Case 2 

A 59-year-old male with relapsed multiple myeloma and cast nephropathy (pretreatment baseline 24-hour urine protein of 0.23 g/d) underwent an anti-BCMA CAR-T therapy in 9/2021, conditioned with lymphocyte-depleting cyclophosphamide and fludarabine chemotherapy. The patient experienced grade I CRS during treatment with a rise in serum creatinine to 2.08 mg/dL, which improved to 1.6 mg/dL 7 days post CAR-T cell infusion. 

In 12/2021, the nephrology service was consulted for progressive proteinuria when a urinalysis showed a rise of total protein to 2.06 g/d persistently from 10/21 through 12/21 without urine Bence Jones protein. Dysmorphic RBCs were found in the urinalysis; schistocytes were seen on the peripheral blood smear (> 2/HPF). The patient experienced worsening thrombocytopenia (nadir of 52,000/μL from a post-treatment baseline of above 100,000/mL). Prothrombin time and partial thromboplastin time (PT/PTT) were normal. Serum fibrinogen was > 600 mg/dL. The patient remained asymptomatic with normal vital signs. Multiple myeloma labs showed non-detectable M-spike and normal kappa/lambda light chain protein ratio in 10/2021. A restaging bone marrow biopsy demonstrated < 1% plasma cell by CD138 immunostaining. 

The significant proteinuria, fragmented RBCs, and the complete remission status of multiple myeloma (MM) suggested the presence of a second glomerular pathology. TMA was thus diagnosed based on clinical grounds, but a kidney biopsy was declined by the patient. The patient continues to be followed clinically and has had a stable course. On 3/2022, 24-hour urine protein was found to be 1.51 g/d; platelet counts returned to a baseline of 100,000. 

## Discussion 

Within the early experience with CAR-T cell therapy, we now present two cases that highly suggest triggering of TMA in the post-CAR-T therapy setting. To our knowledge, no studies have described an association between TMA and CAR-T cell treatment. In both cases, TMA did not occur during or immediately post therapy, when cytokine release should have elicited high levels of complement activation and inflammation. Instead, TMA became clinically apparent 2 – 3 months later with stigmata of MAHA, consumptive thrombocytopenia, and renal injury. In each case, investigation of other potential causes of TMA were negative. Unfortunately, kidney biopsies were not performed due to clinical contraindication and patient choice. In both cases, the clinical course appears to suggest one of slow recovery and stability with continued supportive care. 

How CAR-T cell therapy-associated TMA should be classified poses a challenge as this entity clearly overlaps with TA-TMA in the pathophysiology. With the increasing use of allogeneic CAR-T cells and donor CAR-T cells in the allogeneic transplant settings [4], occurrence of CAR-T-associated TMA will be further confounded by transplant-related causes. Moreover, an accepted definition for TA-TMA currently does not exist. The Bone and Marrow Transplants Clinical Trials Network (CTN) [5] and International Working Group (IWG) [6] have proposed separate criteria for the diagnosis of TA-TMA (Table 1). Cho et al. [7] offered a unified definition of diagnostic criteria for TA-TMA, termed Overall TMA (O-TMA), by integrating the previously proposed diagnostic criteria and omitting the underlying causes. The author defined TMA simply as a known complication of HSCT regardless of its precipitating factor be it graft-versus-host disease, infection, inflammation, or immunosuppressant exposure. Indeed, TA-TMA often occurs in complicated clinical scenarios with multiple potential causes. In our present cases, trigger for TMA may be reasonably explained by preceding CRS or by prior infection. Lymphodepleting chemotherapy has also been identified previously to increase TMA in the transplant setting. Therefore, CAR-T-related TMA may fit more easily into the more pragmatic definition of O-TMA, waiving the difficult task of distinguishing between potential causes of systemic TMA that exist within the overall milieu of CAR-T therapy. Additionally, use of such diagnostic criteria allows for recognition of TMA in the absence of tissue biopsy, which is often prohibited due to thrombocytopenia or medical instability. 

The pathophysiology of TA-TMA has been accepted to be the result of underlying endothelial damage from a variety of insults such as intensive conditioning, irradiation, immunosuppressants, and infection. CAR-T cell therapy has been proposed to cause endothelial damage through cytokines released by microbial translocation across leaky mucosal barriers, the use of immunosuppressive medications, and the process of engraftment [8]. Vascular endothelial cells have also been postulated as a direct tissue target for T lymphocytes, where genetically engineered CAR-T may inflict damage on the endothelial cells [9]. Additionally, other factors encountered in CAR-T therapy including infection, calcineurin inhibitors, and lymphodepleting chemotherapy could all contribute to endothelial injury [10]. 

The timing of endothelial injury in CAR-T therapy is not well understood. Carreras and Diaz-Ricart [8] collected samples of allogenic and auto-HSCT patients, using VWF, ADAMS T-13, and sVCAM-1 as markers of endothelial injury, at various time points post transplant. This study demonstrates peaking of these markers on day 21 post allogeneic transplants and on day 14 post autologous transplants. No studies have investigated time points beyond 30 days using these markers or in CAR-T therapy. Our first patient was more than 12 weeks post CAR-T cell therapy when TMA became clinically apparent. However, both patients were diagnosed within the 100-day window that TA-TMA occurs in experience with allogeneic HSCT [7]. This observation suggests that endothelial injury may be related to ongoing immune-mediated mechanisms that are established later in the post-treatment setting. 

The cumulative incidences of TA-TMA in HSCT have been estimated by Blood and Marrow Transplant-CTN and IWG to be 6.1% and 2.5%, respectively [5, 6]. TA-TMA has been conventionally accepted as a poor prognostic factor in transplant patients. However, both CAR-T therapy patients presently have recovered some of their renal function, suggesting a clinically limited course. The incidence of TMA and its prognostic value in CAR-T therapy should therefore be further investigated with increased use of this treatment in hematopoietic malignancies. 

## Study approval statement 

This study was approved by the University of Washington policies for the Institutional Review Board and HIPAA authorization, as reviewed by the UW Medicine Compliance Policy COMP.103 “Use and Disclosure of Protected Health Information (PHI)”. All HIPAA specific personal health information was removed from the report in accordance with this policy. Approved April 18, 2022. 

## Data availability statement 

All data generated or analyzed during this study are included in this article. Further enquiries can be directed to the corresponding author. 

## Funding 

The authors do not have any funding sources to disclose. 

## Conflict of interest 

The authors do not have any conflict of interests to disclose. 

**Table 1.** Definitions of transplant-associated thrombotic microangiopathy [7].

	O-TMA [7]	CTN-TMA [5]	IWG-TMA [6]
Normal coagulation assays^a^	Yes	Yes	Yes
Schistocytosis	≥ 2/HPF	≥ 2/HPF	≥ 8/HPF
Increase in serum LDH	Yes	Yes	Yes
Concurrent renal^b^ and/or neurologic dysfunction without other explanations		Yes	
Negative Coombs test	Yes	Yes	
Thrombocytopenia^c^	Yes		Yes
Decrease in hemoglobin concentration	Yes		Yes
Decrease in serum haptoglobin	Yes		Yes

LDH = lactate dehydrogenase. ^a^Prothrombin time and activated partial thromboplastin time; ^b^doubling of serum creatinine from baseline or 50% decrease in creatinine clearance from baseline; ^c^de novo, prolonged, or progressive thrombocytopenia (platelet count < 50,000/µL or > 50% reduction.
